# The complete chloroplast genome sequence of *Bromus catharticus* Vahl. (Poaceae)

**DOI:** 10.1080/23802359.2021.1970646

**Published:** 2021-08-31

**Authors:** Li-Ying Feng, Chao Shi, Li-Zhi Gao

**Affiliations:** a Institution of Genomics and Bioinformatics, South China Agricultural University, Guangzhou, China; b Plant Germplasm and Genomics Center, Kunming Institute of Botany, Chinese Academy of Sciences, Kunming, China

**Keywords:** *Bromus catharticus* Vahl., chloroplast genome, Poaceae, phylogeny

## Abstract

*Bromus catharticus* Vahl. belongs to the Pooideae subfamily of Poaceae. In this study, we sequenced and assembled the complete chloroplast genome of *B. catharticus.* The complete chloroplast genome was 134,718 bp in size, including a large single-copy region of 80,540 bp, a small single-copy region of 11,806 bp and a pair of reverse repeats of 21,186 bp in size. The annotation of *B. catharticus* indicates that it contained 89 protein-coding genes, 47 tRNA genes and eight rRNA genes. Our phylogenetic analysis of all protein-coding genes of the 36 grass complete chroloplast genomes using *Cyperus rotundus* as outgroup showed that *B. catharticus* is closely related to the *Koeleria* and *Avena* species to form the Pooideae lineage of the grass family.

*Bromus catharticus* Vahl. belongs to the Pooideae subfamily of Poaceae. It is a winter annual or biennial herb native to South America (Parodi 1947). This grease species often grows on hillsides and is commonly used for short-term forage with high yield and coarse texture (Puecher et al. 2001). *B. catharticus* is a hexaploid species (2*n* = 64) with facultative atresia reproductive behavior (Hauman 1917). Although continuous efforts have been made to reconstruct the phylogeny of Poaceae, phylogenetic relationships have not yet been fully resolved (Saarela et al. 2018). Recently, there has been a rapid progress in comparative chloroplast genomics that has been extensively applied to investigating plant phylogenomics (Huang et al. 2014; Gao et al. 2019). Thus, it is urgently needed to further obtain a large number of grass complete chloroplast genome resources enabling future phylogenomic analyses of the grass family (Gao et al. 2016; Hutang and Gao 2017; Saarela et al. 2018).

In this study, *B. catharticus* plants were collected in the suburbs of Kunming (24°26′30″N, 101°50′25″E), Yunnan Province, China. A specimen was deposited at SCAU (the herbarium of the College of Agriculture, South China Agricultural University https://nxy.scau.edu.cn, Li-zhi Gao, SCAUgenomics@163.com), China, under the voucher number SCAU 2020169. About 20 g fresh mature leaves were sampled from *B. catharticus*, and cpDNAs were extracted by following a modified high salt method reported formerly (Shi et al. 2012). After the cpDNA isolation, approximately 5–10 µg of DNA was sheared, followed by adapter ligation and library amplification, and then subjected to Illumina Sample Preparation Instructions. The fragmented cpDNAs were sequenced at both single-read using the Illumina Genome Analyzer IIx platform at the in-house facility at The Germplasm Bank of Wild Species in Southwestern China, Kunming, China. The obtained paired-end reads (2 × 100 bp read lengths) were assembled using SOAP *de novo* (Li et al. 2010). Regions with ambiguous alignment (conflicted reads mapped to the same genomic region) were trimmed off manually and considered as gaps. Polymerase chain reaction (PCR) amplified fragments yielded by primers derived from the terminal ends of contigs, and the fragments were then sequenced to flank the gap regions. The PCR amplification procedures contained template denaturation at 80 °C for 5 min followed by 30 cycles of denaturation at 95 °C for 30 sec, primer annealing at 55 °C for 30 sec, and primer extension at 65 °C for 1 min; followed by a final extension step of 5 min at 65 °C. PCR products were separated by electrophoresis in 1.5% agarose gel and sequenced on an Applied Biosystems (ABI) 3730 automated sequencer. Subsequently, gene prediction and annotation were performed by DOGMA (Wyman et al. 2004).

The complete chloroplast genome of *B. catharticus* was 134,718 bp in size, comprising two inverted repeat regions (IRs) with a total of 42,372 bp in size, which were split by a large single copy (LSC) with 80,540 bp and small single copy (SSC) with 11,806 bp in length. The chloroplast genome contained 144 functional genes, including 89 protein-coding genes, 47 tRNAs, and eight rRNAs. A total of 21 genes were duplicated in the IR regions, including three rRNA genes (*rrn23*, *rrn4.5*, and *rrn5*), 10 protein-coding genes (*rps19*, *rpl2*, *rpl23*, *ycf2*, *rps7*, *ycf15*, *ndhB*, *ycf68*, *rps15* and *rps12*) and 8 tRNA genes (*trnH-GUG*, *trnI-CAU*, *trnL-CAA*, *trnV-GAC*, *trnI-GAU*, *trnA-UGC*, *trnR-ACG* and *trnN-GUU*). The overall GC content of the *B. catharticus* chloroplast genome was ∼38.44% with the corresponding values of 36.50%, 32.59% and 43.45% in the LSC, SSC, and IR regions, respectively.

To determine the phylogenetic position of *B. catharticus* in the grass family, 35 grass chloroplast genomes together with *Cyperus rotundus* from Cyperaceae were downloaded from GenBank. Phylogenomic analysis was performed by incorporating the *B. catharticus* chloroplast genome obtained in this study. All protein-coding gene sequences were aligned with MAFFT 7409 (Katoh et al. 2002). Using *C. rotundus* as outgroup, phylogenetic tree was reconstructed using the maximum likelihood method using RAxML (Stamatakis 2014) based on 1,000 bootstrap replicates. Our results indicated that the 35 examined grass species were clearly grouped into the twelve subfamilies of Poaceae with strong bootstrap supports (Figure 1). It is apparent that *B. catharticus* is closely related to the *Koeleria* and *Avena* species from Pooideae of the grass family with strong bootstrap supports.

**Figure 1.**
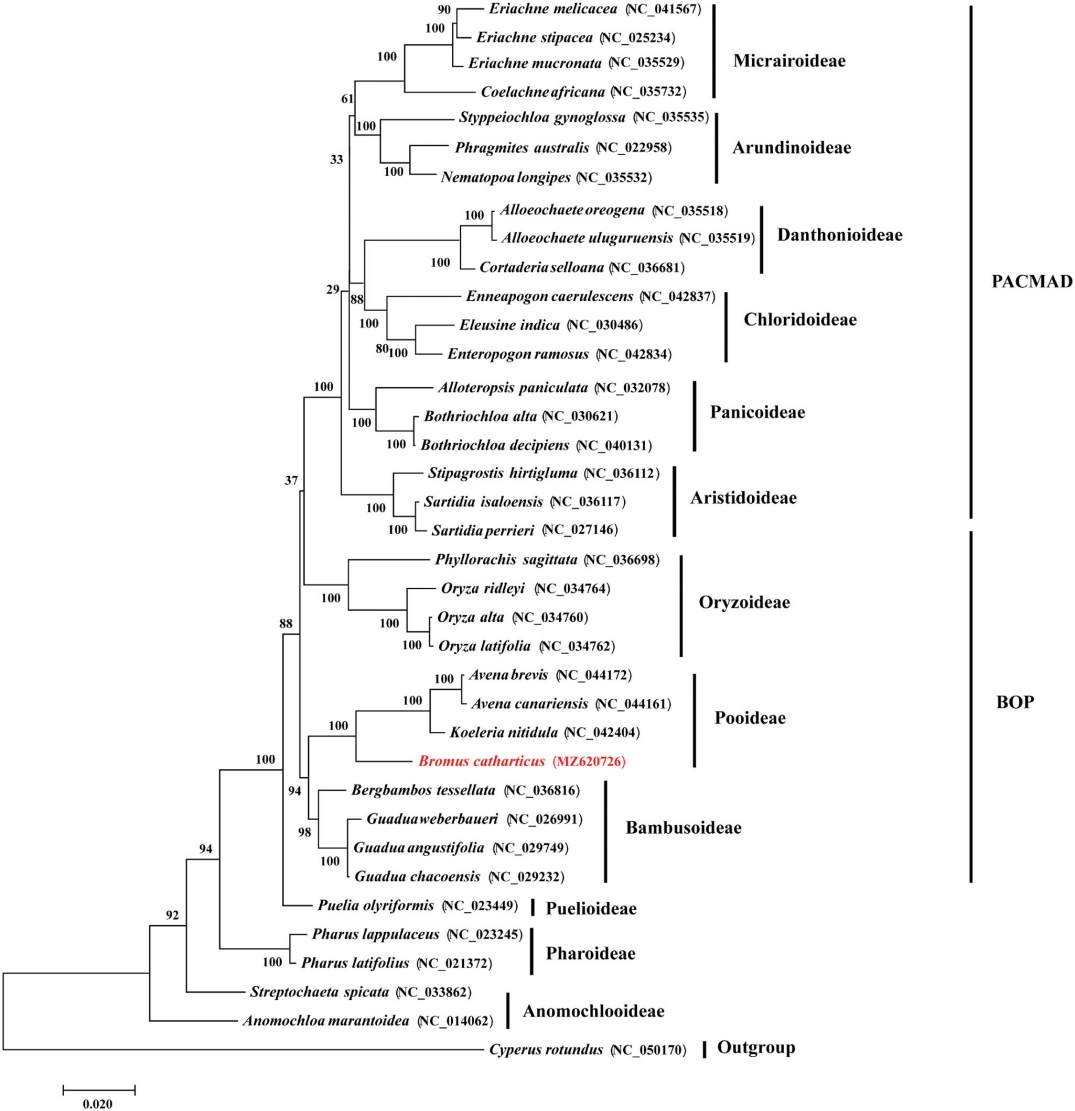
Maximum-likelihood phylogenetic tree based on all protein-coding genes of the 36 grass complete chloroplast genomes using *Cyperus rotundus* as outgroup. Bootstraps values (1,000 replicates) are shown at the nodes.

## Data Availability

The genome sequence data that support the findings of this study are openly available in GenBank of NCBI at [https://www.ncbi.nlm.nih.gov] (https://www.ncbi.nlm.nih.gov/) under the accession MZ620726. The associated BioProject, SRA, and Bio-Sample numbers are PRJNA744348, SRR15234595, and SAMN20166949 respectively. The data that newly obtained at this study are also publicly available in the National Genomics Data Center at https://ngdc.cncb.ac.cn under the accession number of GWHBCHU00000000.

## References

[CIT0001] Gao J, Li K, Gao LZ. 2016. The complete chloroplast genome sequence of the Bambusa multiplex (Poaceae: Bambusoideae). Mitochondrial DNA Part A. 27(2):980–982. 10.3109/19401736.2014.926515 24938112

[CIT0002] Gao LZ, Liu YL, Zhang D, Li W, Gao J, Liu Y, Li K, Shi C, Zhao Y, Zhao YJ, Jiao JY, Mao SY, et al. 2019. Evolution of *Oryza* chloroplast genomes promoted adaptation to diverse ecological habitats. Commun Biol. 2:278. 31372517 10.1038/s42003-019-0531-2 PMC6659635

[CIT0003] Hauman L. 1917. Notes floristiques. Quelques cryptogames, gymnospermes et monocotyledones de l’Argentine. Anales Museo Nac His Nat. 28:391–443.

[CIT0004] Huang H, Shi C, Liu Y, Mao SY, Gao LZ. 2014. Thirteen *Camellia* chloroplast genome sequences determined by high-throughput sequencing: genome structure and phylogenetic relationships. BMC Evol Biol. 14:151. 25001059 10.1186/1471-2148-14-151 PMC4105164

[CIT0005] Hutang GR, Gao LZ. 2017. The complete chloroplast genome sequence of *Leersia perrieri* of the rice tribe Oryzeae (Poaceae). Conserv Genet Resour. 9(4):663–665.

[CIT0006] Katoh K, Misawa K, Kuma K, Miyata T. 2002. MAFFT: a novel method for rapid multiple sequence alignment based on fast fourier transform. Nucleic Acids Res. 30(14):3059–3066. 12136088 10.1093/nar/gkf436 PMC135756

[CIT0007] Li R, Zhu H, Ruan J, Qian W, Fang X, Shi Z, Li Y, Li S, Shan G, Kristiansen K, et al. 2010. *De novo* assembly of human genomes with massively parallel short read sequencing. Genome Res. 20(2):265–272. 20019144 10.1101/gr.097261.109 PMC2813482

[CIT0008] Parodi LR. 1947. Las gramíneas del género *Bromus adventicias* en la Argentina. Rev Arg Agr. 14(1):1–19.

[CIT0009] Puecher DI, Robredo CG, Rios RD, Rimieri P. 2001. Genetic variability measures among *Bromus catharticus* Vahl. populations and cultivars with RAPD and AFLP markers. Euphytica. 121(3):229–236.

[CIT0010] Saarela JM, Burke SV, Wysocki WP, Barrett MD, Clark LG, Craine JM, Peterson PM, Soreng RJ, Vorontsova MS, Duvall MR. 2018. A 250 plastome phylogeny of the grass family (Poaceae): topological support under different data partitions. PEERJ. 6:e4299. 29416954 10.7717/peerj.4299 PMC5798404

[CIT0011] Shi C, Hu N, Huang H, Gao J, Zhao Y, Gao L, Xu Y. 2012. An improved chloroplast DNA extraction procedure for whole plastid genome sequencing. PLOS One. 7(2):e31468. 22384027 10.1371/journal.pone.0031468 PMC3285163

[CIT0012] Stamatakis A. 2014. RAxML version 8: a tool for phylogenetic analysis and post-analysis of large phylogenies. Bioinformatics. 30(9):1312–1313. 24451623 10.1093/bioinformatics/btu033 PMC3998144

[CIT0013] Wyman SK, Jansen RK, Boore JL. 2004. Automatic annotation of organellar genomes with DOGMA. Bioinformatics. 20(17):3252–3255. 15180927 10.1093/bioinformatics/bth352

